# The Impact of Different Retirement Pathways on Cognitive and Physical Health Among Older People in England

**DOI:** 10.1093/geroni/igaf122.1341

**Published:** 2025-12-31

**Authors:** Wei Yang

**Affiliations:** King’s College London, London, England, United Kingdom

## Abstract

Understanding the health implications of retirement is essential for policymaking and aging research. This study explores how different retirement pathways impact cognitive function, mental health, and physical well-being among older adults, drawing on data from the English Longitudinal Study of Ageing (ELSA) wave 1 to wave 10. We apply instrumental variable models with fixed individual and time effects, using state pension age, early retirement, and state pension receipt as instruments. Our findings indicate that retirement generally has a negative impact on cognitive abilities (particularly memory), mental health, and physical functioning in daily life. However, semi-retired individuals—especially those who change employers after reaching retirement—experience better memory, mental health, and physical well-being compared to those who remain fully employed or stay with the same employer. Conversely, full retirement is associated with declines in cognitive function (orientation and memory) and mental health, while having no significant effect on physical health. These results underscore the importance of gradual retirement pathways, enabling older adults to remain engaged in work while mitigating the potential adverse health effects of full retirement.

